# Phytochemical Content, Antioxidant, Alpha-Glucosidase Inhibitory and Antibacterial Activities of Spineless Cactus Pear Cultivars

**DOI:** 10.3390/plants10071312

**Published:** 2021-06-28

**Authors:** Mologadi B. Mabotja, Sonja L. Venter, Christian P. Du Plooy, Tukayi Kudanga, Stephen O. Amoo

**Affiliations:** 1 Agricultural Research Council, Vegetables, Industrial and Medicinal Plants, Private Bag X293, Pretoria 0001, South Africa; MabotjaMB@arc.agric.za (M.B.M.); Sventer@arc.agric.za (S.L.V.); IduPlooy@arc.agric.za (C.P.D.P.); 2 Department of Biotechnology and Food Science, Durban University of Technology, P.O. Box 1334, Durban 4000, South Africa; TukayiK@dut.ac.za; 3 Indigenous Knowledge Systems Centre, Faculty of Natural and Agricultural Sciences, North-West University, Private Bag X2046, Mmabatho 2735, South Africa; 4 Department of Botany and Plant Biotechnology, Faculty of Science, University of Johannesburg, P.O. Box 524, Auckland Park 2006, South Africa

**Keywords:** alpha-glucosidase, *Opuntia ficus-indica*, antibacterial, antioxidant, phytochemicals, diabetes

## Abstract

Variation in cultivars can influence plant biological activities. This study aimed to identify superior cultivars while determining the variability in the phytochemical content, antioxidant, alpha-glucosidase inhibitory and antibacterial activities of cladode extracts from selected spineless Burbank cactus pear (*Opuntia ficus-indica* and *Opuntia robusta*) cultivars. Total phenolic and flavonoid contents were determined using the Folin-Ciocalteu and aluminum chloride spectrophotometric methods, respectively. Antioxidant activity was investigated using 2,2-diphenyl-1-picrylhydrazyl (DPPH) free radical scavenging and β-carotene linoleic acid assays. Alpha-glucosidase inhibition was determined using a spectrophotometric method and antibacterial activity using a non-polar (petroleum ether) and polar (50% methanol) extracts against two Gram-positive and two Gram-negative bacteria. Significant variation in phytochemical content, antioxidant, antidiabetic and antibacterial activities was observed amongst the cultivars. Alpha-glucosidase inhibitory activity varied widely with IC_50_ values ranging from 0.06 to 1.85 mg/mL. Radical scavenging activity of Polypoly cultivar was about seven fold higher than that recorded in other cultivars with low activity. Turpin and Berg x Mexican cultivars had the highest total phenolic and flavonoid contents, whilst the non-polar extract of Turpin also exhibited higher antibacterial activity against *Bacillus subtilis* and *Escherichia coli*. Sicilian Indian Fig was amongst the cultivars with a higher antioxidant activity, whilst also showing a strong inhibition against *B. subtilis* and *E. coli*. Polypoly cultivar demonstrated strong antioxidant and antidiabetic activities while its polar extract showed the highest total antibacterial activity against *B. subtilis*. The cultivar Malta was superior in terms of its antibacterial potency and efficacy against *B. subtilis, Staphylococcus aureus* and *E. coli*. The potential of using spineless cactus pear cladodes as a functional food with antioxidant, antidiabetic and antibacterial properties against pathogenic food spoilage bacteria in place of synthetic compounds was established. The significance of cultivar selection to increase this potential was highlighted.

## 1. Introduction

Lifestyle changes, poor nutrition and exposure to hazardous conditions, amongst others, have a negative impact on public health, as reflected in an increase in chronic diseases such as diabetes, cancer, obesity and hypertension [1]. According to the International Diabetes Federation (IDF) [2], diabetes is among the most common non-communicable diseases globally. Diabetes was rated as the fourth leading cause of death in most developed countries [3]. It is a lifetime progressive metabolic disease and is the most common endocrine disease that has affected an estimated 9.3% of the worldwide adult population [4]. The two common types of diabetes include Type 1, arising from the inability of the pancreatic β-cells to produce insulin, and Type 2, which is caused by insulin resistance and/or insufficient insulin production [1].

An increase in new and re-emerging pathogens with severe cases of antibiotic resistance remains a global concern. Resistance to current drugs, insufficient/incompatible therapies and negative side effects associated with some currently used drugs, amongst others, have rendered some management practices of diabetes and some infectious diseases almost ineffective [5,6]. All of these issues favor the growing interest in the use of herbal remedies for the treatment of diabetes and infectious diseases, as they are perceived to have a high economic value and fewer side effects when compared to synthetic agents [5,6]. Foodborne pathogenic bacteria such as *Staphylococcus aureus*, *Bacillus subtilis* and *Escherichia coli* have biofilm-forming abilities, causing food deterioration or spoilage, which is another global health dilemma [7,8]. There is an increase in research activities to find new alternative and strong antimicrobial agents, particularly from plants [9].

The use of *Opuntia* spp. (family Cactaceae), a climate-smart plant, as a remedy for diabetes and infectious diseases, has been documented from as early as the 1970′s, with *Opuntia ficus-indica* (L.) Mill. cladodes frequently used for the treatment of type-2 diabetes in Mexico [10]. *Opuntia* spp., commonly known as cactus or prickly pear, is well known for its multipurpose use, particularly as a food source and for medicinal purpose. Cactus pear cladodes contain beneficial and therapeutic phytochemicals that exhibit a vast variety of pharmacological activities including antioxidant, hypoglycemic and hypocholesterolemic activities, as well as protective effects against chronic diseases such as cancer, diabetes, and cardiovascular diseases [11,12]. Unlike the spiny cactus pear, which is highly invasive and is classified as a weed in South Africa, the spineless cultivars are relatively easy to manage and are cultivated as a climate-smart crop in South Africa, especially its cladodes, as animal fodder [13]. Different cactus pear cultivars contain several phytochemicals including antioxidants such as ascorbic acid, carotenoids, taurine, cysteine, reduced glutathione and flavonoids, such as kaempferol, quercetin and isorhamnetin [13,14]. The protective effect of cladode extracts against oxidative damage was mainly attributed to different antioxidants including vitamin E, ascorbic acid, carotenoids, flavonoids and phenolic acids [15,16]. Cactus pear cladode extracts also exhibited a potential growth inhibition of multi-drug-resistant food and human pathogens associated with skin infections, food contamination and nosocomial infections [17]. For example, Sánchez et al. [18] observed antimicrobial activities of *Opuntia ficus-indica* cladode methanol extracts from eight cultivars against *Campylobacter jejuni*, *Vibrio cholera*, and *Clostridium perfringens* [18]. Other studies indicated an antibacterial activity of cactus pear cladode methanolic extracts against *Enterococcus faecium*, *E. coli*, *Salmonella* spp., *Pseudomonas aeruginosa*, and *S. aureus* [19,20]. However, research has indicated that factors including cultivar type, plant age and environmental conditions can influence plant bioactive compound concentrations and biological activities [21,22]. For example, a comparative study indicated higher flavonol and phenolic contents in two South African cultivars compared to some Sicilian and Egyptian cultivars [22]. Another study showed variations in the antioxidant and antibacterial activities of extracts from eight cactus pear cultivars [18]. The aim of this study was to identify superior cultivars while determining the variability in the phytochemical content, antioxidant, alpha-glucosidase inhibitory and antibacterial activities of cladode extracts from selected spineless Burbank cactus pear (*Opuntia ficus-indica* and *Opuntia robusta* J.C. Wendl.) cultivars.

## 2. Results and Discussion

### 2.1. Cladode Extraction Yield

Two solvents (50% methanol and petroleum ether) with different polarities were used for extraction and the yields of the resulting crude extracts from both solvents are presented in Table 1. The yields varied amongst the cultivars in both solvent extractions. In general, extraction with 50% (*v*/*v*) methanol (MeOH) gave higher yields (ranging from 6.75 to 26.10% *w*/*w*) as compared to petroleum ether (ranging from 0.14 to 1.88% *w*/*w*). The higher yields recorded with 50% MeOH extracts may be due to the fact that polar solvents extract polar compounds, which are in a higher abundance than non-polar compounds that are extracted by a non-polar solvent (petroleum ether) [23]. The highest extract yields were recorded in cultivar Santa Rossa for methanol extracts and Muscatei for petroleum ether extracts, which were three-fold and thirteen-fold of the lowest yields for methanol and petroleum ether extracts, respectively.

### 2.2. Total Phenolic and Flavonoid Contents

**Figure 1:**
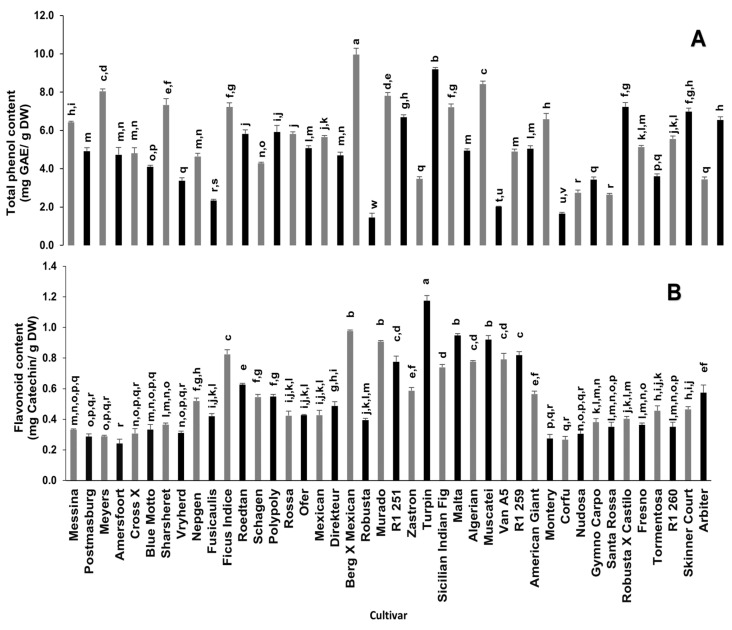
Total phenolic (**A**) and flavonoid (**B**) contents of cladodes from 42 spineless cactus pear cultivars. Bars bearing different letters in each graph are significantly different (*p* = 0.05) according to Duncan’s Multiple Range Test (DMRT). Values are mean ± standard errors (n = 3). GAE—Gallic Acid Equivalents.

Phenolic compounds play a significant role in the antioxidant potential of various plants due to their redox properties, which allow them to act as reducing agents, hydrogen donors, singlet oxygen quenchers, and metal chelators [11]. Significant differences in total phenolic and flavonoid contents amongst the different cultivars were observed (Figure 1). Berg x Mexican and Turpin cultivars had significantly high total phenolic contents (9.96 mg gallic acid equivalent (GAE)/g dry weight (DW) and 9.19 mg GAE/g DW, respectively), which were five times higher when compared to the Robusta cultivar with the lowest total phenolic content (1.5 mg GAE/g DW). Similarly, the flavonoid content of Turpin (1.17 mg catechin equivalent (CE)/g DW) and Berg x Mexican (0.98 mg CE/g DW) cultivars were approximately four-fold of the cultivars with a low flavonoid content (Amersfoort, Postmasburg, Meyers, Cross X, Vryherd, Montery, Corfu and Nudosa), ranging from 0.24 to 0.31 mg CE/g DW.

A study by du Toit et al. [13] on the dried cladodes of five of the cultivars used in the current study reported a total phenolic content ranging from 0.18–0.27 mg/g, which was lower than those recorded in this study. Furthermore, the total phenolic content of the cultivars used in this study is higher than that of some Mexican cultivars [18] and some Brazilian cultivars [24]. Both the Mexican cultivars [18] and those used by du Toit et al. [13] were 6 months old and the Brazilian cultivars [24] were 3 years old as compared to the one-year-old cultivars used in this study. Conversely, the total phenolic content in the current study was low in comparison to that of some varieties (wild varieties of blanco, cristalino, morado and tempranillo) reported by Guevara-Figueroa et al. [25] Similarly, the flavonoid content in the current study, which ranged from 0.24 to 1.17 mg CE/g DW, was low when compared to the study of Sánchez et al. [18] who reported a flavonoid content ranging from 15.4 to 36.6 mg quercetin equivalent/g dry weight. Guevara-Figueroa et al. [25] also reported a high flavonoid content of 9.8 and 5.9 mg quercetin equivalent/g dry weight for the blanco and manso commercial varieties, respectively. Nonetheless, the total phenolic content in the current study was higher than the flavonoid content [25], in contrast to the report of Sanchez et al. [18]. Cladode maturity and/or environmental conditions can influence bioactive compound concentrations [25,26] and this fact may explain the variance observed in our study when compared to some other studies.

### 2.3. Antioxidant Activity of Cladode Extracts

**Figure 2:**
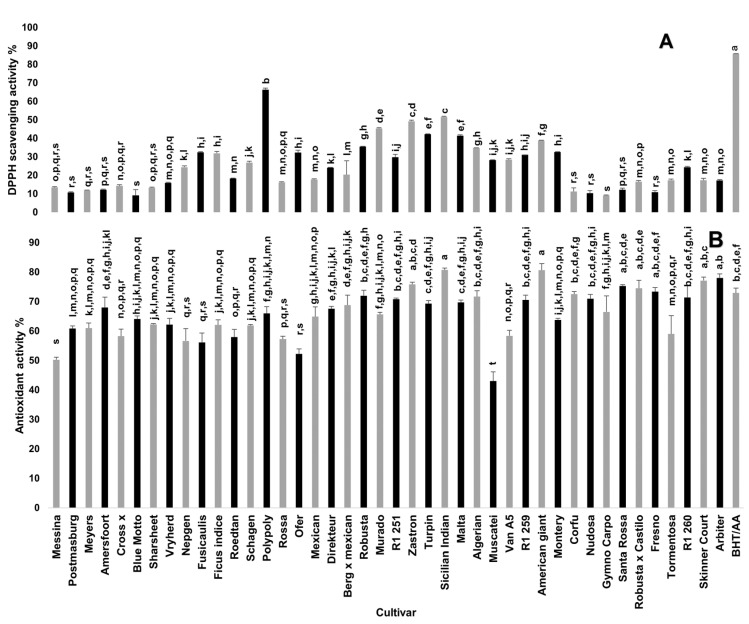
Antioxidant activity of cladode methanolic extracts from 42 spineless cactus pear cultivars. (**A**) DPPH free radical scavenging activity (%) at 10 mg/mL extract concentration. (**B**) Antioxidant activity (%) based on bleaching rate in β-carotene-linoleic acid assay. Bars bearing different letters in each graph indicate significant differences (*p* = 0.05) according to Duncan’s Multiple Range Test (DMRT). Values are means ± standard errors (n = 3). Butylated hydroxytoluene (BHT) was used as a standard for total antioxidant activity while ascorbic acid (AA) was used as a standard for DPPH free radical scavenging activity.

Figure 2 shows the free radical scavenging and antioxidant activities of cladode methanolic extracts from 42 spineless cactus pear cultivars. A statistically significant variation was observed among the cultivars. Polypoly cultivar showed the highest free radical scavenging activity (66.37%), which was approximately seven-fold what was recorded for cultivars with low free radical scavenging activity including Messina, Postmasburg, Meyers, Amersfoort, Fresno, Gymno Carpo, Blue Motto and Corfu. A previous study [27] indicated free radical scavenging activity ranging from 83.77–95.53% in cladodes of cactus pear cultivars, which is higher than that recorded in this study (9.1–66.37%). Haile et al. [28] recorded the radical scavenging activity of cactus pear cladodes that ranged from 59.3–85.8%. The concentrations at which the extracts were evaluated in each study may be a confounding factor in the results reported. Nevertheless, the current study indicated variations in the cultivar antioxidant activity.

The inclusion of antioxidant agents, particularly from natural sources such as plants, is of great importance in the cosmetic and food industry [29]. Although antioxidants are essential for protecting cells against free radicals, it is important to have a balanced system of oxidants and antioxidants [30]. Oxidants present at acceptable levels play an important role in the production of new skin cells by initiating cell-signaling pathways, resulting in the removal of UV-damaged cells [31].

Due to the complex mechanism of action of antioxidants, the use of at least two different assays has become standard practice [32]. Antioxidant activity based on β-carotene-linoleic acid assay of the cultivars ranged from 43 to 80%. The highest antioxidant activity (80%), which was significantly higher than that of butylated hydroxytoluene (BHT), was recorded in Sicilian Indian Fig and American giant cultivars whilst the Muscatei cultivar had the lowest activity (43%). Twenty-three of the cultivars exhibited antioxidant activity comparable to that of BHT. BHT is a synthetic antioxidant usually used as a food additive to prevent the damage caused by free radicals during the oxidation processes [33]. The use of BHT as a food additive has potential health hazards for consumers including long-term toxic effects on the liver and lungs [34,35]. In addition to their antioxidant potential as food additives, natural antioxidants such as cactus pear cladodes may be used as a food colorant and could positively influence the sensory characteristics of stabilized food [36]. The cladodes of selected spineless cactus pear cultivars (such as Polypoly, Sicilian Indian Fig and American giant), which demonstrated high in vitro antioxidant activities in this study, can be used in the food industry as nutraceutical and functional foods, and potentially in the cosmetic industry to protect against reactive oxygen species that cause skin disorders [37].

### 2.4. Alpha-Glucosidase Inhibitory Activity

Table 2 presents the alpha-glucosidase inhibitory activity of the cladode extracts. The extracts significantly inhibited alpha-glucosidase with IC_50_ values ranging from 0.06 to 1.85 mg/mL. The lower the IC_50_, the stronger the inhibitory activity. Of the 42 cultivars investigated, 27 cultivars exhibited stronger alpha-glucosidase inhibitory activity with IC_50_ values significantly lower than that of acarbose (1.07 mg/mL), which is a widely used drug for the treatment of type-2 diabetes [38]. Eleven of the cultivars (Berg X Mexican, Blue Motto, Cross X, Ficus Indice, Messina, Nepgen, Ofer, Polypoly, Postmasburg, Roedtan and Sharsheret) demonstrated alpha-glucosidase inhibitory activity (IC_50_ < 0.1 mg/mL) that was 10-fold stronger) when compared to acarbose. Alpha-glucosidase is a known key enzyme in carbohydrate digestion and its inhibition is considered as a therapeutic target for the modulation of postprandial hyperglycemia, a common abnormality in type-2 diabetes [39]. Alpha-glucosidase inhibitors can be effective in the management of hyperglycemia by delaying the effects of postprandial hyperglycemia [40]. The noteworthy in vitro alpha-glucosidase activity of the cladode extracts indicates their potential use as a functional food in the effective management of diabetes, even if it varies due to cultivar.

### 2.5. Correlation Analysis of Phytochemical Content, Antioxidant and Alpha-Glucosidae Inhibitory Activities

Significantly, positive and moderate correlations were established between the total phenolic and flavonoid contents, flavonoid content and DPPH radical scavenging activity, as well as between antioxidant and antidiabetic activities (Table 3). This observation is in line with previous studies demonstrating the positive roles of flavonoids and phenolic compounds in free radical scavenging [11,13] as well as the roles of antioxidants, particularly via inhibition of lipid peroxidation in the treatment or management of diabetes [41,42,43,44]. Direct selection of cultivars with a high flavonoid content may result in increased free radical scavenging while selection for cultivars with strong antioxidants (through inhibition of lipid peroxidation) may lead to increased antidiabetic properties. This finding is also significant in cultivar selection during breeding programs.

### 2.6. Antibacterial Activity

The antibacterial activities of extracts from selected cultivars against two Gram-positive and two Gram-negative bacteria are presented in Table 4. The lower the minimum inhibitory concentration (MIC) values, the stronger the antibacterial activity in terms of potency. In general, some noteworthy antibacterial activities were observed against *B. subtilis* and *E. coli* with MIC values below 1 mg/mL [45]. Weak activity was observed against *K. pneumoniae*.

Low antibacterial activity against Gram-negative bacteria may be due to the thick murein layer in their structure preventing the entry of inhibitors [46]. Poor activity in some of the extracts may be due to low concentrations of antibacterial compounds in the extracts [47]. The differential antibacterial activity of *Opuntia dillenii* (Ker Gawl.) Haw. extracts against two Gram-negative bacteria, *E. coli* and *Salmonella typhi*, has also been reported [48].

Petroleum ether extracts generally had better antibacterial activity in terms of potency, when compared to 50% methanol extracts. This is mostly associated with the difference in polarity of the solvents. Umar et al. [48] similarly observed a considerably improved antimicrobial activity against Gram-positive *B. subtilis* and *S. aureus* with non-polar extracts of *O. dillenii* when compared with polar extracts. Similar observations regarding the superior activity/potency of non-polar extracts against both Gram-positive and Gram-negative bacteria have been reported [49,50]. In terms of antibacterial potency, the best MIC value was recorded with petroleum ether extract of Malta cultivar (0.39 mg/mL) against *B. subtilis* and *E. coli*, and 0.78 mg/mL against *S. aureus*. Overall, the cultivars demonstrating potent antibacterial activity (MIC < 1.0 mg/mL) against at least two bacteria are Malta, Roedtan, Sicilian Indian Fig and Turpin whereas Murado and R1251 demonstrated potent antibacterial activity against one bacterium (*B. subtilis* and *E. coli*, respectively). In a similar study, methanol, ethanol, and chloroform extracts of *Opuntia ficus-indica* cladodes exhibited considerable antibacterial activity against *Streptococcus pneumoniae*, *S. typhi*, *B. subtilis* and *E. coli* [9]. In addition, Kim et al. [19,20] reported antibacterial activity of *Opuntia ficus-indica* methanol extracts against *Enterococcus faecium, E. coli*, *Salmonella* spp., *Pseudomonas aeruginosa*, and *S. aureus*.

Total activity (or minimum inhibitory dilution), which is the volume to which the bioactive compounds in one gram can be diluted and still inhibit bacteria growth, provides a measure of antibacterial efficacy while the minimum inhibitory concentration indicates potency [51,52,53]. Table 5 presents the total antibacterial activity of the cladode extracts against the bacteria evaluated in this study. The polar (methanol) extract demonstrated better efficacy than the non-polar (petroleum ether) extract, owing to the higher extraction yield with the polar extract. At least one extract of the cultivars Malta, Polypoly and R1 251 showed the highest total activity against one of the bacteria. Therefore, in terms of both antibacterial potency and efficacy of the selected 20 cultivars, the cultivar Malta demonstrated superior antibacterial activity. Moreover, the strong antibacterial activity of some of the spineless cladodes documented in this study suggests the potential use of spineless cladodes against food spoilage or pathogenic microorganisms, although a careful cultivar selection may be required.

## 3. Materials and Methods

### 3.1. Plant Material Collection and Preparation

Cladodes were collected from one-year old 42 spineless cactus pear cultivars grown under the same glasshouse conditions at the Agricultural Research Council, Roodeplaat Research farm, South Africa (25°36′1″ S 28°21′42″ E). Two cultivars (Robusta and Monterey) belonged to *Opuntia robusta* while the remaining cultivars were from *Opuntia ficus-indica*. Each cultivar consisted of five plants and selection was done on good quality cladodes with no bruises or discoloration. For each cultivar, cladodes from the first, third and fifth plant were harvested. The harvested cladodes were sliced into small pieces and oven-dried at 50 °C in the dark. The material was then ground into fine powder using a pulverizing mill. To determine the percentage yield of extracts from the cladodes of 42 spineless cactus pear cultivars, 20 g dry weight (DW) of each cultivar was extracted separately with 300 mL of 50% (*v*/*v*) methanol and petroleum ether in order to obtain methanolic (MeOH) and petroleum ether (PE) extracts, respectively. Each mixture (plant material and solvent) was sonicated for an hour in an ultrasonic water bath (Branson, 5510E-MT, Lasec, South Africa) and filtered through Whatman No.1 filter paper, followed by in vacuo concentration using a rotary evaporator (Stuart, RE300DB, Lasec, South Africa) at 40 °C and air-drying in a fume hood.

### 3.2. Total Phenolic and Flavonoid Content Determination

The extraction procedure was carried out as described by Amoo et al. [54] Plant material (0.2 g) was extracted by sonication in an ultrasonic bath containing ice-cold water for 30 min using 10 mL of 50% MeOH, followed by centrifuging at 1073.3× *g* for 2 min. Total phenolic content was determined using the Folin-Ciocalteu method [55], with modifications. A reaction mixture containing 50 µL of the sample extract, 450 µL distilled water, 250 µL of Folin-Ciocalteu reagent and 1250 µL sodium carbonate (2% *w*/*v*) was briefly vortexed and incubated for 40 min at room temperature. Thereafter, absorbance was recorded using a spectrophotometer (Specord 210 plus, Analytik Jena, Jena, Germany) at 725 nm. The assay was done in triplicate and a calibration curve was prepared using gallic acid as a standard. Results were expressed in mg gallic acid equivalent (GAE) per gram dry weight (DW).

Flavonoid content was determined using an aluminum chloride method [56], with modifications. A reaction mixture containing 250 µL sample extract, 1.6 mL of distilled water, 75 µL (5% *w*/*v*) sodium nitrite, 75 µL of aluminum chloride (10% *w*/*v*), and 0.5 mL (1 M) NaOH was briefly vortexed and absorbance measured at 510 nm. The assay was done in triplicates and a calibration curve was prepared using catechin as a standard. Results were expressed in mg catechin equivalent (CE) per gram DW.

### 3.3. Antioxidant Assays

An approach into antioxidant investigation of natural compounds can be a strenuous process due to their diverse chemical structures, biological roles and different modes of actions [57]. Hence, different antioxidant procedures may give different results because each assay has its own thermodynamics and kinetics [32]. A selection of reliable antioxidant procedures that measure different properties such as radical scavenging, phase distribution equilibria, proton and electron transfer, and relate to food and biological systems is of importance in antioxidant investigation [32].

#### 3.3.1. DPPH (2,2-diphenyl-1-picrylhydrazyl) Free Radical Scavenging Activity

Samples were extracted using the method described previously by Amoo et al. [54], with slight modifications. An amount of 20 g dried powdered cladode from each cultivar was extracted with 300 mL of 50% MeOH by sonication for 1 h. The extract volume was condensed on a rotary evaporator at 40 °C before air-drying. The antioxidant activity was determined using the DPPH method [58], with modifications [53]. At different known concentrations, 30 µL of 50% MeOH extracts were diluted with 720 µL MeOH followed by an addition of 750 µL DPPH solution. Ascorbic acid was used as a positive control. The mixture was incubated at room temperature (25 ± 2 °C) for 40 min before recording absorbance at 517 nm. The assay was done in triplicate and the percentage free radical scavenging activity (RSA) was calculated using Equation (1):

(1) $$RSA\;\left(\%\right)=\left\{\;1-\left(\frac{Abs_{517\;nm}\;\;Sample-Abs_{517\;nm}\;Blank\;\;}{Abs_{517\;nm}\;Neg\;Control}\right)\right\}\times 100$$

where *Abs*_517_ nm; Sample is the absorbance of the sample mixture; *Abs*_517_ nm Neg Control is the absorbance of the negative control (MeOH); and *Abs*_517_ nm Blank is the absorbance of the blank (50% MeOH in place of DPPH).

#### 3.3.2. Antioxidant Activity Using β-Carotene Linoleic Acid Assay

Following the extraction procedure described above in Section 3.3, antioxidant activity using β-carotene linoleic acid assay was determined [53,59]. An aliquot (2.4 mL) of β-carotene emulsion consisting of β-carotene (5 mg) dissolved in chloroform (1 mL), linoleic acid (100 µL), Tween 20 (1 mL), and distilled water (248 mL) was dispensed into reaction tubes containing 100 µL of sample extract at a predetermined concentration. Butylated hydroxytoluene was prepared as a positive control at 6.25 mg/mL. Aqueous methanol (50%) in place of the sample was used as a negative control. The assay was conducted in triplicates. Absorbance was measured at 470 nm immediately and then a second absorbance reading at 470 nm was done after incubation in a water bath at 50 °C for 1 h. β-carotene bleaching rate was calculated using Equation (2):

(2) $$Bleaching\;rate\;\left(R\right)=\left\{\ln\left(\frac{A_{t=0}}{A_{t=t}}\right)\right\}\times \frac{1}{t}$$

where *A*_*t*=0_ is the absorbance of the emulsion at 0 min and *A_t=t_* is the absorbance of the emulsion at 60 min. The bleaching rate was used to calculate the percentage antioxidant activity (ANT) expressed as a percentage inhibition of the rate of β-carotene bleaching using Equation (3):

(3) $$\%\;ANT=\left(\;\frac{R{\;}_{control}-R_{\;sample}}{R{\;}_{control}}\right)\times 100$$

where *R_control_* and *R_sample_* are the average β-carotene bleaching rates for the control and plant extract or BHT, respectively.

### 3.4. Alpha-Glucosidase Inhibitory Activity

Alpha-glucosidase inhibitory activity was determined using a method described by Li et al. [60], with modifications. Yeast alpha-glucosidase (0.5 unit/mL) was dissolved in 0.1 M potassium phosphate buffer (pH 6.8) and the substrate (5 mM *p*-nitrophenyl-α-d-glucopyranoside) was prepared in the same buffer (pH 6.8). Different concentrations of the samples were prepared using dimethyl sulfoxide (DMSO). Sample wells contained 20 µL sample, 100 µL 0.1 M potassium phosphate buffer (pH 6.8), and 20 µL yeast alpha-glucosidase (0.5 unit/mL) enzyme solution. Sample blank wells contained 20 µL sample, 100 µL 0.1 M potassium phosphate buffer (pH 6.8) and 20 µL DMSO. Negative control wells contained 20 µL DMSO, 100 µL 0.1 M potassium phosphate buffer (pH 6.8), and 20 µL yeast alpha-glucosidase (0.5 unit/mL) enzyme solution. The plates were pre-incubated at 37 °C for 5 min after which 20 µL of the substrate was added to initialize the reaction. After further incubation at 37 °C for 30 min, the reaction was stopped by adding 80 µL of 0.2 M sodium carbonate (prepared in the same potassium phosphate buffer). The tests were performed in triplicates and acarbose was used as a positive control. The amount of *p*-nitrophenol (pNP) released was quantified using a 96-well microplate reader at 405 nm. The alpha-glucosidase inhibitory rate (%) was calculated using Equation (4).

(4) $$\%\;inhibition\;rate=[1-\left(\;\frac{Abs{\;}_{sample}-Abs_{\;sample\;blank}}{Abs{\;}_{negative\;control}}\right)]\times 100$$

where *Abs_sample_* is the absorbance of the sample mixture, *Abs_sample blank_* is the absorbance of Sample blank and *Abs_negative control_* is the absorbance of the negative control.

### 3.5. Antibacterial Activity

After preliminary experiments, twenty cultivars were selected based on their total phenolic and flavonoid content (seven of the highest, six intermediate and seven of the lowest content) and further profiled for their antibacterial activity. Antibacterial activity was determined using a serial micro-plate dilution assay [61]. Extracts were tested against two Gram-positive—*Staphylococcus aureus* (ATCC 9144), *Bacillus subtilis* (ATCC 6051)—and two Gram-negative—*Escherichia coli* (ATCC 8739) and *Klebsiella pneumonia* (ATCC 13883)—bacteria. The bacterial cultures were maintained on Mueller Hinton agar medium in petri dishes and an inoculum of each microorganism was grown in Mueller Hinton broth and incubated at 37 °C for 24 h. An equal volume (100 µL) of distilled water and plant extract was transferred into first row wells and two-fold serially diluted through the 96-well plates to prepare extracts with different concentrations. A hundred microliters of the bacterial solution were then added to all the wells and ciproflaxin was used as a positive control. For negative control, 50% MeOH and petroleum ether were used. The plates were incubated for 24 h at 37 °C. After incubation, 40 µL of *p*-iodonitrotetrazolium chloride (INT) was added and minimum inhibitory concentration (MIC) values were determined as the lowest concentration where there was no color change. Bacterial growth was indicated by pink color, whilst bacterial inhibition was indicated by no color change after addition of INT.

### 3.6. Data Analysis

The IC_50_, which is the concentration of the extract required to inhibit 50% of the alpha-glucosidase, was determined for each extract using GraphPad Prism software (version 4.03). The data were log-transformed, normalized, and fitted into a nonlinear regression for IC_50_ determination. Data were subjected, as appropriate, to one-way analysis of variance using Statsoft (Statistica 8) software. The mean values were compared based on Duncan’s multiple range test and a significant difference was established at *p* = 0.05. Pearson correlation coefficient analysis was computed using SPSS software (version 16) and significant correlation was established at *p* ≤ 0.05.

## 4. Conclusions

The significant influence of cultivar when using cactus pear as a potential functional and nutraceutical food product was highlighted in this study. Strong antidiabetic activity coupled with the observed antioxidant and antibacterial activities, although varied with cultivars, indicate the potential of using cladodes as a functional food and in applications against food spoilage in place of synthetic compounds. Although the cultivars exhibited different levels of activity, some cultivars are superior in terms of their phytochemical content and/or biological properties. Turpin and Berg x Mexican had both the highest phenolic and flavonoid content, whilst the non-polar extract of Turpin also exhibited a higher antibacterial activity against *B. subtillis* and *E. coli*. Sicilian Indian Fig was amongst the cultivars with a higher antioxidant activity whilst also showing great inhibition against *B. subtillis* and *E. coli*. Polypoly was among the superior cultivars showing strong antioxidant and antidiabetic activities, while its polar extract showed the highest total antibacterial activity against *B. subtilis*. The non-polar extract of Malta demonstrated superior antibacterial activity in terms of both potency and efficacy against three bacteria (*B. subtilis*, *S. aureus*, and *E. coli*). The cultivars with high phytochemical contents and biological activities have potential to be used as food additives against food spoilage and in the fight against diabetes and pathogenic organisms.

## Figures and Tables

**Table 1:** Percentage yield of extracts from cladodes of 42 spineless cactus pear cultivars.

Cultivar	Extract Yield (% *w*/*w*)		Cultivar	Extract Yield (% *w*/*w*)	
	50% Methanol	Petroleum Ether		50% Methanol	Petroleum Ether
Algerian	21.30	0.33	Nudosa	19.66	0.19
American giant	6.75	0.76	Ofer	15.24	0.19
Amersfoort	15.95	0.80	Polypoly	14.28	0.14
Arbiter	11.97	0.89	Postmasburg	13.00	0.48
Berg x Mexican	21.10	0.32	R1 251	22.50	0.55
Blue Motto	18.63	0.41	R1 259	13.63	0.22
Corfu	8.57	0.25	R1 260	18.86	0.21
Cross x	22.04	0.88	Robusta	15.79	0.78
Direkteur	15.03	0.24	Robusta x Castilo	17.59	0.57
Ficus indice	17.38	0.18	Roedtan	9.29	0.23
Fresno	13.46	0.47	Rossa	25.14	0.79
Fusicaulis	13.16	0.52	Santa Rossa	26.10	0.58
Gymno Carpo	20.22	0.21	Schagen	12.41	0.69
Malta	25.38	0.95	Sharsheet	19.39	0.97
Messina	14.74	0.86	Sicilian Indian fig	11.81	0.18
Mexican	10.36	0.22	Skinner Court	15.13	0.35
Meyers	23.83	0.88	Tormentosa	11.93	0.20
Montery	17.83	0.63	Turpin	22.05	0.23
Murado	17.78	0.22	Van A5	9.65	0.37
Muscatei	20.17	1.88	Vryherd	10.13	0.65
Nepgen	20.27	0.73	Zastron	13.04	0.46

**Table 2:** Alpha-glucosidase inhibitory activity of cladode extracts from 42 spineless cactus pear cultivars.

Cultivar	IC_50_ (mg/mL)	Cultivar	IC_50_ (mg/mL)
Algerian	0.52 ± 0.002 ^b^	Nudosa	1.43 ± 0.017 ^l,m^
American Giant	1.11 ± 0.075 ^f,g,h^	Ofer	0.09 ± 0.003 ^a^
Amersfoort	0.10 ± 0.001 ^a^	Polypoly	0.08 ± 0.001 ^a^
Arbiter	0.96 ± 0.021 ^e^	Postmasburg	0.06 ± 0.000 ^a^
Berg x Mexican	0.08 ± 0.000 ^a^	R1 251	0.89 ± 0.004 ^d,e^
Blue Motto	0.09 ± 0.000 ^a^	R1 259	1.50 ± 0.002 ^m^
Corfu	1.85 ± 0.165 ^n^	R1 260	1.21 ± 0.024 ^h,i,j^
Cross X	0.07 ± 0.003 ^a^	Robusta	0.10 ± 0.000 ^a^
Direkteur	0.10 ± 0.002 ^a^	Robusta X Castilo	1.19 ± 0.003 ^g,h,i^
Ficus Indice	0.07 ± 0.004 ^a^	Roedtan	0.07 ± 0.002 ^a^
Fresno	1.35 ± 0.088 ^k,l^	Rossa	0.11 ± 0.004 ^a^
Fusicaulis	0.10 ± 0.000 ^a^	Santa Rossa	1.30 ± 0.007 ^i,j,k^
Gymno Carpo	1.37 ± 0.007 ^k,l^	Schagen	0.12 ± 0.001 ^a^
Malta	0.85 ± 0.008 ^d^	Sharsheret	0.09 ± 0.001 ^a^
Messina	0.08 ± 0.000 ^a^	Sicilian Indian Fig	1.12 ± 0.013 ^f,g,h^
Mexican	0.13 ± 0.003 ^a^	Skinner Court	1.30 ± 0.007 ^j,k^
Meyers	0.11 ± 0.000 ^a^	Tormentosa	1.11 ± 0.120 ^f,g,h^
Montery	1.09 ± 0.001 ^f,g,h^	Turpin	1.09 ± 0.004 ^f,g^
Murado	0.12 ± 0.001 ^a^	Van A5	0.66 ± 0.025 ^c^
Muscatei	1.44 ± 0.003 ^l,m^	Vryherd	0.10 ± 0.000 ^a^
Nepgen	0.08 ± 0.000 ^a^	Zastron	0.74 ± 0.004 ^c^
		* Acarbose	1.07 ± 0.058 ^f^

Mean values with different letters indicate significant differences (*p* = 0.05) according to Duncan’s Multiple Range Test. Values are means ± standard errors (n = 3). * Acarbose was used as a positive control.

**Table 3:** Pearson correlation coefficient analysis for the phytochemical, antioxidant, and antidiabetic properties of cladodes from the 42 spineless cactus pear cultivars.

Parameters	Total Phenolic	Flavonoid	DPPH ^#^	Antioxidant ^§^
Total phenolic	1.00			
Flavonoid	0.45 **	1.00		
DPPH	0.15	0.58 **	1.00	
Antioxidant	0.17	0.05	0.18	1.00
Antidiabetic	−0.20	0.09	−0.04	0.46 **

^#^ DPPH = 2,2-diphenyl-1-picrylhydrazyl free radical scavenging activity; ^§^ Antioxidant = Antioxidant activity based on β-carotene linoleic acid assay. ** = *p* ≤ 0.05.

**Table 4:** Antibacterial activity (minimum inhibitory concentration; mg/mL) of cladodes from 20 selected spineless cactus pear cultivars.

Cultivar	*Bacillus subtilis*		*Staphylococcus aureus*		*Escherichia coli*		*Klebsiella pneumoniae*	
	MeOH	P.E.	MeOH	P.E.	MeOH	P.E.	MeOH	P.E.
Algerian	6.25	1.56	>6.25	3.13	>6.25	3.13	3.13	3.13
American Giant	>6.25	3.13	3.13	3.13	3.13	>6.25	>6.25	>6.25
Berg X Mexican	6.25	1.56	>6.25	3.13	>6.25	1.56	3.13	>6.25
Blue Motto	>6.25	1.56	>6.25	>6.25	3.13	1.56	6.25	>6.25
Corfu	3.13	>6.25	3.13	>6.25	3.13	>6.25	>6.25	>6.25
Direkteur	>6.25	1.56	3.13	1.56	6.25	1.56	>6.25	>6.25
Fresno	>6.25	>6.25	>6.25	1.56	>6.25	1.56	6.25	>6.25
Gymno Carpo	6.25	3.13	>6.25	1.56	>6.25	>6.25	3.13	>6.25
Malta	3.13	**0.39**	6.25	**0.78**	>6.25	**0.39**	>6.25	3.13
Mexican	6.25	1.56	>6.25	1.56	1.56	1.56	6.25	>6.25
Murado	>6.25	**0.78**	>6.25	1.56	>6.25	1.56	>6.25	>6.25
Nudosa	3.13	6.25	6.25	1.56	>6.25	>6.25	3.13	>6.25
Ofer	6.25	1.56	3.13	1.56	6.25	1.56	3.13	>6.25
Polypoly	1.56	1.56	>6.25	1.56	3.13	1.56	3.13	>6.25
R1 251	6.25	1.56	3.13	1.56	1.56	**0.78**	3.13	1.56
R1 259	6.25	3.13	6.25	3.13	>6.25	>6.25	>6.25	>6.25
Roedtan	>6.25	**0.78**	>6.25	1.56	6.25	**0.78**	6.25	>6.25
Sicilian Indian Fig	>6.25	**0.39**	>6.25	1.56	>6.25	**0.78**	>6.25	>6.25
Tormentosa	>6.25	3.13	>6.25	1.56	6.25	1.56	6.25	6.25
Turpin	>6.25	**0.78**	3.13	1.56	>6.25	**0.78**	>6.25	>6.25
* Ciproflaxin	0.10		0.10		0.05		0.10	

Bold values indicate MIC values considered as good activity (MIC < 1 mg/mL). * Ciproflaxin was used as a positive control (mg/mL). MeOH—Methanol. P.E.—Petroleum ether.

**Table 5:** Total antibacterial activity (minimum inhibitory dilution; mL/g) of cladode extracts from 20 selected spineless cactus pear cultivars.

Cultivar	*Bacillus subtilis*		*Staphylococcus aureus*		*Escherichia coli*		*Klebsiella pneumoniae*	
	MeOH	P.E.	MeOH	P.E.	MeOH	P.E.	MeOH	P.E.
Algerian	34.1	2.12	ND	1.05	ND	1.05	68.05	1.05
American Giant	ND	2.43	21.57	2.43	21.57	ND	ND	ND
Berg X Mexican	33.76	2.05	ND	1.02	ND	2.05	67.41	ND
Blue Motto	ND	2.63	ND	ND	59.52	2.63	29.81	ND
Corfu	27.38	ND	27.38	ND	27.38	ND	ND	ND
Direkteur	ND	1.54	48.02	1.54	24.05	1.54	ND	ND
Fresno	ND	ND	ND	3.01	ND	3.01	21.54	ND
Gymno Carpo	32.35	0.67	ND	1.35	ND	ND	64.60	ND
Malta	81.09	**24.36**	40.61	**12.18**	ND	**24.36**	ND	3.04
Mexican	16.58	1.41	ND	1.41	66.41	1.41	16.58	ND
Murado	ND	2.82	ND	1.41	ND	1.41	ND	ND
Nudosa	62.81	0.30	31.46	1.22	ND	ND	62.81	ND
Ofer	24.38	1.22	48.69	1.22	24.38	1.22	48.69	ND
Polypoly	**91.54**	0.90	ND	0.90	45.62	0.90	45.62	ND
R1 251	36.00	3.53	**71.88**	3.53	**144.23**	7.05	**71.88**	**3.53**
R1 259	21.81	0.70	21.81	0.70	ND	ND	ND	ND
Roedtan	ND	2.95	ND	1.47	14.86	2.95	14.86	ND
Sicilian Indian Fig	ND	4.62	ND	1.15	ND	2.31	ND	ND
Tormentosa	ND	0.64	ND	1.28	19.09	1.28	19.09	0.32
Turpin	ND	2.95	70.45	1.47	ND	2.95	ND	ND

ND—not determined. Bold values indicate the highest total activity per extract per bacterium. MeOH—Methanol. P.E.—Petroleum ether.

## Data Availability

All data related to this study are already presented in this publication.
